# Glanders in Newark, N. J.

**Published:** 1884-01

**Authors:** 


### GLANDERS IN NEWARK, N. J.

An outbreak of glanders and farcy in the stables of the Newark and South Orange Horse-car R.R. Company has caused considerable excitement in Newark and vicinity during the last two months. This excitement grew the more intense, because the President (and virtual owner) of the line refused in a most obstinate manner to check the progress of this dreadful disease, and yielded only after recourse was had by the local Board of Health to the most stringent measures available under the existing rather complicated and deficient laws on the subject. The obstinacy of the owner of the affected horses was partly due to the advice of an "ignorant" graduate and to the opinions of other so-called veterinarians who failed to recognize the true nature of the disease when it first appeared in the stables. It is somewhat remarkable that the parties who most emphatically stated that the disease was not glanders all disagreed in their diagnosis. One called it purpura hæmorra hagica, another, strangles; a third, nasal gleet, etc., etc. When rumors of the existence of glanders in the South Orange car stables reached the Newark Board of Health steps were immediately taken to check its spread, the company doing almost its entire business in the city. The local Board has on its staff a veterinarian, who was immediately ordered to make an examination, and upon his reporting the true nature of the disease Dr. Mandeville, the health physician, displayed a most commendable energy in co-operating with the veterinarian to prevent its spread. The great difficulty, however, was the fact that the stables were located outside of the city limits and therefore out of the jurisdiction of the Board. Another difficulty was the regretful circumstances that, although the State Board of Health, which by law possessed the necessary powers, failed to act as promptly as the occasion required, though it had been duly notified. The local Board complying with a provision of the State law, caused a notice to kill the diseased animals to be served upon Mr. Rädel, and issued by the senior police justice of Newark, who is also a justice of the peace and therefore has jurisdiction in the county. But Mr. Rädel paid no attention to the notice and even threatened to bring suit should any of his horses be killed by the health authorities. At last strategy had to be resorted to and the Mayor of Newark, who is President of the Board of Health, notified Mr. Rädel, that if he did not comply with the requests of the Board he would revoke the license of all cars of the company. This remedy worked like a charm and Mr. Rädel surrendered. Under supervision of the authorities one mule and fifty-six horses were killed and all suspicious animals quarantined. The premises were thoroughly disinfected and a large amount of other property destroyed. I should like to state here that the law under which Mr. Rädel's horses were killed is a very unjust one, inasmuch as it does not provide for any compensation, which to some extent is due to the unfortuate owner, who, for the public good, is obliged to suffer a serious loss at the hands of the State. This defect ought to be remedied by the next Legislature. The post mortem examations made on the carcasses revealed glanders and farcy in all its various stages. In about fourteen cases the septum nasi was completely destroyed. After his surrender Mr. Rädel stated that the first horse affected came into his stables in the earlier part of last January. In February a number of horses were diseased and some of them died. The disease then spread from month to month, with an increasing rate of mortality, about forty or more horses having died up to the time of the interference by the authorities. As could be expected the owner of the horses on account of his stubbornness, and his veterinary ignoramus were severely and universally condemned by the press and public. The State Board of Health was censured for its dilatory action in the matter. The Newark Board of Health, and especially Dr. Mandeville, the Health Physician, are justly entitled to praise, for it is due to their energetic action that the outbreak was localized and an epidemic prevented which might have caused loss of human life.

"VETERINARIAN."

